# Ramen restaurant prevalence is associated with stroke mortality in Japan: an ecological study

**DOI:** 10.1186/s12937-019-0482-y

**Published:** 2019-09-04

**Authors:** Kosuke Matsuzono, Makiko Mieno, Shigeru Fujimoto

**Affiliations:** 1 Division of Neurology, Department of Medicine, Jichi Medical University School of Medicine, Yakushiji 3311-1, Shimotsuke, Tochigi 329-0498 Japan; 2 Department of Medical Informatics, Center for Information, Jichi Medical University School of Medicine, Yakushiji 3311-1, Shimotsuke, Tochigi 329-0498 Japan

**Keywords:** Stroke epidemiology, Ramen, Japanese diet, Risk factor, Culture

## Abstract

**Background:**

The association between stroke and nutrition has recently been investigated. However, the association between diet and stroke in Japan has not been clarified. We hypothesized that there may be an association between consumption of ramen and stroke mortality. Therefore, we investigated the association between the prevalence of ramen restaurants and stroke mortality in Japanese prefectures.

**Methods:**

We used Pearson’s correlation coefficients to evaluate associations between the prevalence of each of four restaurant types (ramen, fast food, French or Italian, and udon or soba) and age- and sex-adjusted stroke mortality rates in each prefecture. We also investigated correlations between acute myocardial infarction and the prevalence of each type of restaurant as a control. We obtained age- and sex-adjusted stroke mortality rates and the acute myocardial infarction mortality rate in each prefecture from the *2017 Trends in National Health* published in Japan. Data on the number of restaurants of each type in each prefecture were obtained from the database of the Nippon Telegraph and Telephone Corporation.

**Results:**

The prevalence of ramen restaurants, but not of other restaurant types, positively correlated with stroke mortality in both men and women (*r* > 0.5). We found no correlation between ramen restaurant prevalence and mortality from acute myocardial infarction.

**Conclusion:**

The prevalence of ramen restaurants in Japanese prefectures has a significant correlation with the stroke mortality rate.

**Electronic supplementary material:**

The online version of this article (10.1186/s12937-019-0482-y) contains supplementary material, which is available to authorized users.

## Background

Stroke is a major cause of death and disability worldwide and its association with nutrition has been investigated recently [1]. Healthy diets, such as the Mediterranean diet and the Dietary Approaches to Stop Hypertension (DASH) diet, have been shown to reduce the risk of stroke [2–4], as has higher protein intake [5]. In contrast, higher consumption of carbohydrates or salt increases stroke risk and mortality [6, 7]. Japanese diets, which tend to be high in salt and low in animal-derived fatty acids, have been reported to reduce cardiovascular mortality [8]. However, stroke morbidity and mortality remain high in Japan and the reasons for the lack of association between stroke and coronary heart disease outcomes have not been determined [9]. In addition, stroke mortality in Eastern Japan is higher than that in Western Japan. Cultural differences, especially differences in food preferences, may be associated with regional disparities in stroke mortality; however, the underlying causes remain unknown.

Approximately 40 years ago, stroke was the leading cause of death in Japan [9]. However, the age-adjusted stroke mortality rate has since markedly decreased compared with that of coronary heart disease. A key factor in this improvement is thought to be changing lifestyles [10]. The traditional Japanese diet was thought to increase stroke risk because of the heavy use of seasonings such as miso, salt, and soy sauce. However, associations between type of diet and stroke have not been examined in Japan. Ramen is one of the most popular foods in Japan, despite being of Chinese origin [11]. Since its original introduction in Japan, ramen has been adapted and now consists of wheat noodles served in broth topped with sliced pork, seaweed, or menma (a Japanese condiment made from lacto-fermented bamboo shoots; Additional file 1). Being tasty and inexpensive, ramen became a popular food that was available from street food stands in Japan after World War ΙΙ. Although the number of ramen stands has decreased markedly, ramen remains highly popular in Japan. High dietary sodium content was recently reported to be a risk factor for stroke [12]; ramen has a high sodium content. However, the relationship between stroke and ramen consumption remains unclear. In this study, we investigated the association between the number of ramen restaurants in each Japanese prefecture and stroke mortality in that prefecture.

## Methods

### Study design

We determined the prevalence of ramen, fast food, French or Italian, and udon or soba restaurants in each prefecture in Japan. We then analyzed correlations between the prevalence of each type of restaurant and the age- and sex-adjusted stroke and acute myocardial infarction (AMI) mortality rates. The Ethics Committee of Jichi Medical University approved this study (approval #Rin-Dai 17–147); the Institutional Review Board waived the requirement for consent of participants or patients for this study.

### Study cohort and restaurant prevalence

We obtained age- and sex-adjusted stroke mortality rates and the AMI mortality rate for each prefecture from the *2017 Trends in National Health*, published by the Health, Labour and Welfare Statistics Association in Japan. Data on the number of restaurants of each type in each prefecture were obtained from the database of the Nippon Telegraph and Telephone Corporation (http://itp.ne.jp/) on 21 December 2017.

### Map creation

We used the free software Shirochizu-Nurinuri (3kaku-K) (https://n.freemap.jp/) to create a map showing the age- and sex-adjusted stroke mortality rates and the prevalence of ramen restaurants in each prefecture in Japan.

### Statistical analysis

We calculated the Pearson’s correlation coefficient for the prevalence of each type of restaurant (ramen, fast food, French or Italian, and udon or soba) and age- and sex-adjusted stroke and AMI mortality rates. All statistical analyses were performed with JMP 10 statistical software (SAS Institute, Cary, NC, USA); *P* < 0.05 was considered to denote statistical significance. We evaluated correlations with the correlation coefficient (*r*) value.

## Results

**Fig. 1:**
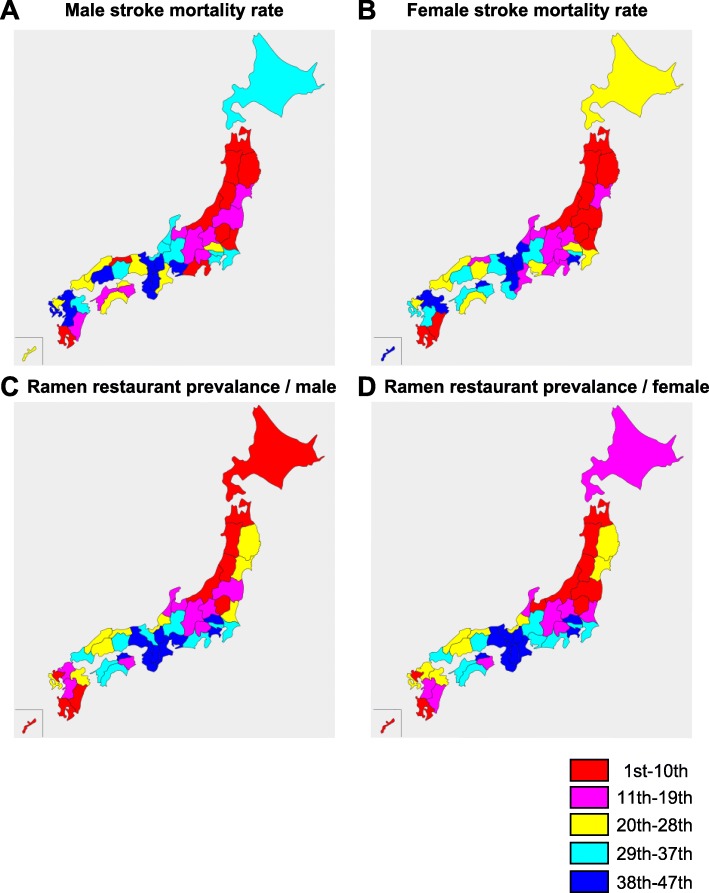
Maps showing age- and sex-adjusted stroke mortality rates and the prevalence of ramen restaurants in each Japanese prefecture. Red indicates high rate; blue indicates low rate. **a** Age-adjusted stroke mortality rate among men. **b** Age-adjusted stroke mortality rate among women. **c** Ramen restaurant prevalence per male resident. **d** Ramen restaurant prevalence per female resident

We analyzed the relationship between the prevalence (the number of restaurants divided by the number of people living in each prefecture) of ramen, fast food, French or Italian, and udon or soba restaurants and age- and sex-adjusted stroke mortality rates. Our results indicate that the Tohoku region (especially the area adjacent to the Sea of Japan), the northern Kanto region, and the southern Kyushu region have high stroke mortality (Fig. 1). In contrast, the Kinki region and southern Kanto region have low stroke mortality rates. This stroke mortality distribution corresponded approximately with the prevalence of ramen restaurants in these regions.

**Fig. 2:**
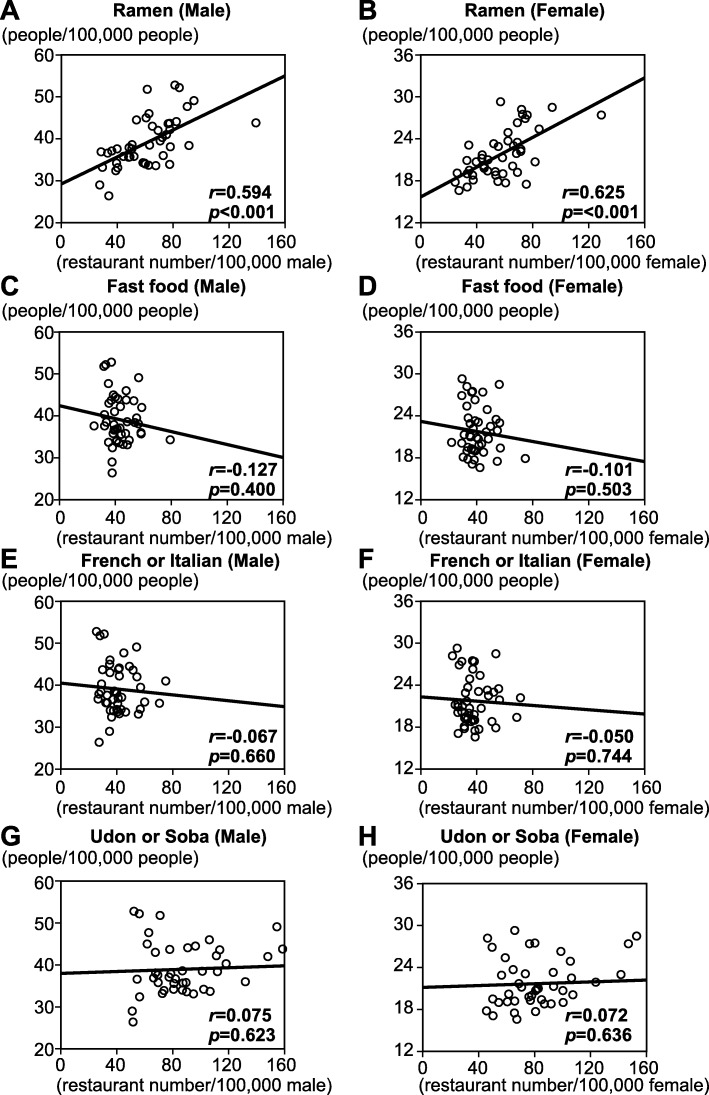
Scatter plots of age-adjusted stroke mortality rates for men and women and the prevalence of the four types of restaurant in each prefecture

**Fig. 3:**
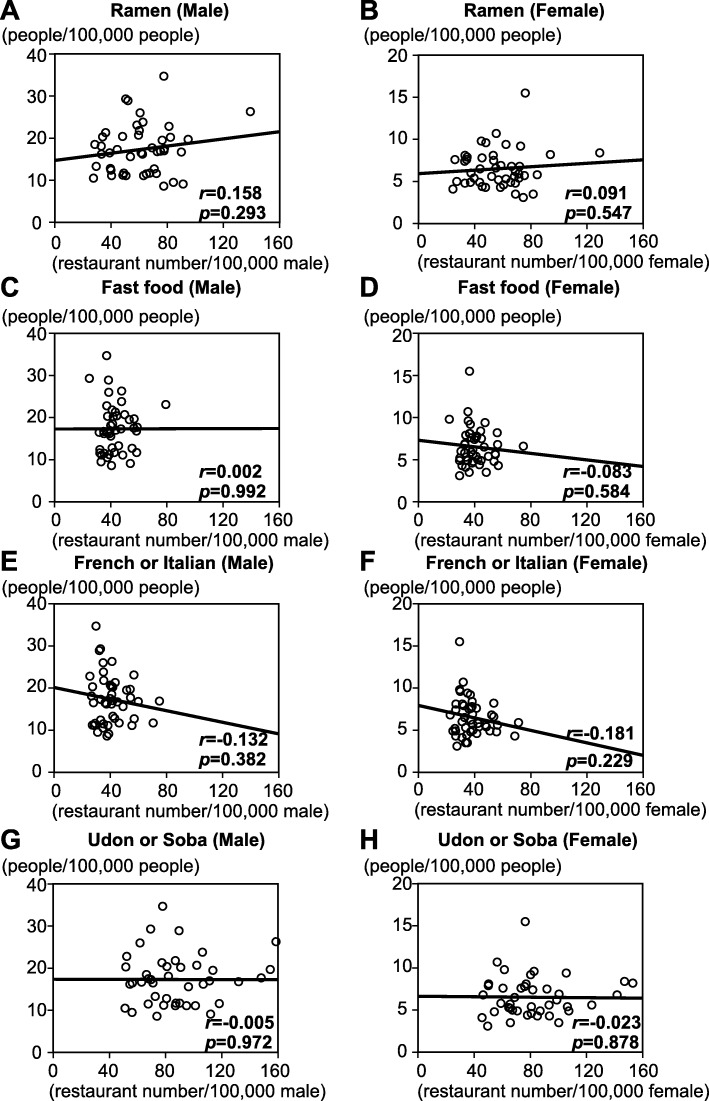
Scatter plots of age- and sex-adjusted acute myocardial infarction mortality rates of men and women and the prevalence of the four types of restaurant in each prefecture

The scatter plots of the relationship between age- and sex-adjusted stroke mortality rates and the prevalence of each restaurant type (Fig. 2) showed that ramen restaurants were the only restaurant type for which there was a positive correlation (*r >* 0.5) between restaurant prevalence and stroke mortality rate. This correlation was present for both men (*r =* 0.594) and women (*r =* 0.625). In contrast, the scatter plots showed no significant correlations between age- and sex-adjusted AMI mortality rates and the prevalence of any restaurant type (Fig. 3). The raw data are shown in Additional files 2 and 3.

## Discussion

Among the types of restaurants examined, only the prevalence of ramen restaurants was positively correlated with age- and sex-adjusted stroke mortality rates. We acknowledge that we have not identified a direct relationship between ramen consumption and stroke mortality rates. Accurate analysis of individual diets was not possible because there is no public research database of specific kinds of food consumed. In addition, there are several types of orders offered in ramen restaurants (e.g., noodles, tsukemen) as well as instant noodles prepared at home. However, the prevalence of a specific type of restaurant is controlled by local demand and therefore provides some indication of regional food preferences. In addition, our results help to explain the relatively high stroke mortality in the northern Kanto and southern Kyushu regions of Japan. Ischemic stroke has a seasonal pattern in Japan, with ischemic stroke in winter associated with a poor prognosis [13]. Both low and high seasonal temperatures have been reported to increase the stroke mortality rate; however, the mechanisms underlying these trends are unclear [14]. Our findings suggest that diet preferences may explain regional differences in stroke mortality rates. Our study also revealed that both the prevalence of ramen restaurants and the stroke mortality rate are low in the Kinki region and in the southern Kanto region. We speculate that the factors influencing this result are as follows: 1) Restaurants with more extensive offerings than those of ramen restaurants are needed for business functions; 2) encouragement of a healthy diet is relatively common in urbanized cities such as Tokyo, Yokohama, Osaka, and Kyoto, which are located in these regions; and 3) historically, it was not necessary to preserve food using salt in these regions, which are located near the sea.

Relationships between diet and health have been reported previously. French and Italian cuisine is mainly consistent with the Mediterranean diet, which is generally considered a model of a healthy diet [15]. Most udon and soba restaurants serve both udon and soba. Rutin, a bioflavonoid found in soba, has been shown to have neuroprotective effects in some stress experiments [16], which suggests that soba has the potential to be part of a healthy diet. Fast food is high in unhealthy fats, salt, and sugar and contributes to obesity [17]. However, the prevalence of fast food restaurants was not a risk factor for stroke mortality in our study. We speculate that only a limited subgroup consumes fast food frequently in Japan.

It is important to emphasize that our study outcome was stroke mortality, not stroke morbidity. Hemorrhagic and cardioembolic strokes are subtypes with a poor prognosis [18, 19]. Certain characteristics of ramen (high temperature, high salt, and high carbohydrate content) may be of particular concern regarding hemorrhagic or cardioembolic stroke; however, data supporting this possibility are lacking.

Our study had several limitations. First, we did not directly assess whether ramen intake increases stroke risk. Consumption of ramen is not restricted to ramen restaurants. For example, we did not consider the impact of consumption of home-cooked instant ramen in our study. Second, there are many components of ramen, including soy, tonkotsu (a Japanese broth made from simmered pork marrow or pork bone), miso, and salt. The types or components of ramen that most strongly affect stroke risk remain unclear. Third, ramen restaurants do not serve only ramen; they also serve various side dishes such as dumplings and rice. These side dishes may include confounding nutritional factors. Finally, the mechanisms by which ramen intake increases stroke risk remain unclear.

## Conclusions

Ramen, a popular food in Japan, is high in carbohydrates and salt and thus may increase the risk of stroke mortality. Our findings indicate a correlation between the regional prevalence of ramen restaurants and stroke mortality.

## Additional files

Additional file 1: Typical ramen appearance. (PDF 45193 kb)

Additional file 2: Male age-adjusted stroke or acute myocardial infarction mortality (AMI) rates and the prevalence of the four types of restaurant in each prefecture. (DOC 78 kb)

Additional file 3: Female age-adjusted stroke or acute myocardial infarction mortality (AMI) rates and the prevalence of the four types of restaurant in each prefecture. (DOC 78 kb)

## Data Availability

The dataset supporting the conclusions of this article is included within the article and additional files.

## Abbreviations

AMI: acute myocardial infarction
DASH: Dietary Approaches to Stop Hypertension
