# Supplementary figures and images for: Ramen restaurant prevalence is associated with stroke mortality in Japan: an ecological study
Source: Nutr J. 2019 Sep 4;18:53. doi: 10.1186/s12937-019-0482-y (PMC6727387; doi:10.1186/s12937-019-0482-y)

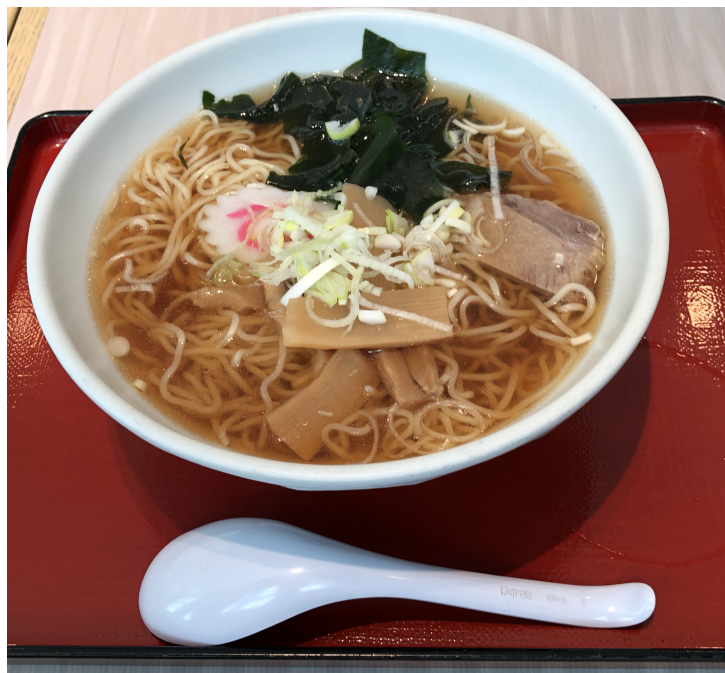

Supplement: Supplementary file 1 — Typical ramen appearance. (PDF 45193 kb) [file 12937_2019_482_MOESM1_ESM.pdf]
