# Supplementary material for: Ramen restaurant prevalence is associated with stroke mortality in Japan: an ecological study
Source: Nutr J. 2019 Sep 4;18:53. doi: 10.1186/s12937-019-0482-y (PMC6727387; doi:10.1186/s12937-019-0482-y)
Supplement: Supplementary file 2 — Male age-adjusted stroke or acute myocardial infarction mortality (AMI) rates and the prevalence of the four types of restaurant in each prefecture. (DOC 78 kb) [file 12937_2019_482_MOESM2_ESM.doc]

Male age-adjusted stroke or acute myocardial infarction mortality (AMI) rates and the prevalence of the four types of restaurant in each prefecture

| Prefecture name | Age-adjusted stroke mortality rate (male) | Age-adjusted AMI mortality rate (male) | Restaurant kinds | | | |
| --- | --- | --- | --- | --- | --- | --- |
|  |  |  | Ramen | Fast food | French  or Italian | Udon  or Soba |
|  | (people/100,000male) | | (restaurant number/100,000male) | | | |
| Hokkaido | 34.7 | 14.5 | 77.6 | 39.3 | 33.1 | 70.8 |
| Aomori | 52.8 | 22.8 | 81.3 | 37.2 | 25.5 | 52.3 |
| Iwate | 51.8 | 16.5 | 61.5 | 31.8 | 28.0 | 71.0 |
| Miyagi | 43.0 | 11.5 | 65.1 | 35.4 | 35.1 | 67.6 |
| Akita | 52.2 | 9.5 | 84.4 | 33.1 | 31.0 | 56.1 |
| Yamagata | 43.8 | 26.3 | 139.2 | 47.8 | 41.2 | 158.6 |
| Fukushima | 43.7 | 34.7 | 77.6 | 37.1 | 29.9 | 77.9 |
| Ibaragi | 46.0 | 23.8 | 62.7 | 47.7 | 35.2 | 106.2 |
| Tochigi | 49.1 | 19.7 | 94.9 | 56.6 | 54.2 | 154.5 |
| Gunma | 39.5 | 12.7 | 70.8 | 55.2 | 57.1 | 165.4 |
| Saitama | 36.7 | 18.1 | 33.0 | 40.0 | 26.7 | 82.0 |
| Chiba | 35.8 | 17.3 | 44.4 | 47.3 | 33.2 | 69.6 |
| Tokyo | 35.7 | 11.7 | 49.1 | 58.4 | 70.6 | 87.4 |
| Kanagawa | 36.6 | 16.2 | 33.2 | 39.1 | 37.3 | 54.5 |
| Niigata | 47.7 | 16.7 | 90.1 | 35.0 | 45.1 | 62.9 |
| Toyama | 43.6 | 19.5 | 76.4 | 53.2 | 51.7 | 113.6 |
| Ishikawa | 36.0 | 16.8 | 72.9 | 58.3 | 60.0 | 132.0 |
| Fukui | 34.3 | 23.1 | 58.4 | 79.2 | 56.8 | 175.8 |
| Yamanashi | 42.0 | 17.7 | 69.2 | 58.9 | 54.4 | 148.0 |
| Nagano | 41.0 | 16.9 | 75.2 | 39.4 | 75.0 | 198.4 |
| Gifu | 35.6 | 20.4 | 48.2 | 44.2 | 40.7 | 80.9 |
| Shizuoka | 44.5 | 15.6 | 53.8 | 40.0 | 49.1 | 96.3 |
| Aichi | 34.2 | 12.8 | 39.5 | 41.0 | 43.1 | 80.7 |
| Mie | 37.1 | 21.3 | 36.1 | 43.3 | 43.5 | 77.4 |
| Shiga | 26.4 | 20.3 | 34.0 | 37.7 | 27.4 | 51.6 |
| Kyoto | 33.1 | 11.1 | 40.4 | 48.6 | 55.5 | 94.9 |
| Osaka | 33.2 | 13.3 | 29.4 | 45.6 | 41.9 | 72.6 |
| Hyogo | 36.9 | 18.5 | 28.5 | 41.3 | 41.1 | 66.4 |
| Nara | 29.0 | 10.5 | 27.5 | 37.7 | 34.8 | 51.1 |
| Wakayama | 32.4 | 16.5 | 39.0 | 37.9 | 36.3 | 56.3 |
| Tottori | 45.0 | 26.0 | 60.7 | 38.6 | 34.9 | 61.8 |
| Shimane | 38.5 | 11.1 | 63.1 | 33.5 | 28.7 | 101.2 |
| Okayama | 35.8 | 28.9 | 52.0 | 38.4 | 32.2 | 89.6 |
| Hiroshima | 33.7 | 16.2 | 62.4 | 35.1 | 40.9 | 106.8 |
| Yamaguchi | 37.9 | 11.2 | 48.5 | 38.9 | 27.3 | 78.6 |
| Tokushima | 40.3 | 11.6 | 72.3 | 32.2 | 29.1 | 118.2 |
| Kagawa | 37.6 | 12.4 | 39.9 | 32.3 | 33.1 | 214.0 |
| Ehime | 38.6 | 11.1 | 50.6 | 48.5 | 38.3 | 86.9 |
| Kochi | 37.6 | 29.3 | 50.4 | 24.8 | 33.0 | 69.3 |
| Fukuoka | 33.6 | 11.7 | 67.6 | 42.6 | 46.3 | 90.0 |
| Saga | 38.4 | 9.1 | 91.3 | 54.0 | 38.9 | 112.0 |
| Nagasaki | 34.0 | 21.8 | 60.1 | 41.0 | 35.4 | 86.9 |
| Kumamoto | 33.9 | 8.6 | 77.5 | 40.6 | 37.7 | 73.8 |
| Oita | 34.2 | 20.7 | 59.6 | 49.9 | 40.1 | 102.2 |
| Miyazaki | 42.2 | 17.0 | 77.7 | 43.6 | 41.9 | 111.2 |
| Kagoshima | 44.1 | 20.2 | 82.3 | 41.8 | 41.8 | 90.8 |
| Okinawa | 38.1 | 17.5 | 78.0 | 56.5 | 40.0 | 67.8 |

AMI: acute myocardial infarction
