# Supplementary material for: Ramen restaurant prevalence is associated with stroke mortality in Japan: an ecological study
Source: Nutr J. 2019 Sep 4;18:53. doi: 10.1186/s12937-019-0482-y (PMC6727387; doi:10.1186/s12937-019-0482-y)
Supplement: Supplementary file 3 — Female age-adjusted stroke or acute myocardial infarction mortality (AMI) rates and the prevalence of the four types of restaurant in each prefecture. (DOC 78 kb) [file 12937_2019_482_MOESM3_ESM.doc]

Female age-adjusted stroke or acute myocardial infarction mortality (AMI) rates and the prevalence of the four types of restaurant in each prefecture

| Prefecture name | Age-adjusted stroke mortality rate (female) | Age-adjusted AMI mortality rate (female) | Restaurant kinds | | | | |
| --- | --- | --- | --- | --- | --- | --- | --- |
|  |  |  | Ramen | Fast food | French  or Italian | | Udon  or Soba |
|  | (people/100,000female) | | (restaurant number/100,000female) | | | | |
| Hokkaido | 21.0 | 5.5 | 69.2 | 35.1 | 29.5 | 63.0 | |
| Aomori | 28.2 | 6.8 | 72.0 | 32.9 | 22.6 | 46.4 | |
| Iwate | 29.3 | 5.2 | 57.0 | 29.5 | 26.0 | 65.8 | |
| Miyagi | 23.7 | 5.3 | 62.3 | 33.8 | 33.6 | 64.7 | |
| Akita | 26.9 | 3.1 | 74.6 | 29.3 | 27.4 | 49.6 | |
| Yamagata | 27.4 | 8.4 | 129.1 | 44.3 | 38.2 | 147.1 | |
| Fukushima | 27.4 | 15.5 | 76.0 | 36.4 | 29.3 | 76.4 | |
| Ibaragi | 24.9 | 9.4 | 62.3 | 47.4 | 34.9 | 105.6 | |
| Tochigi | 28.5 | 8.2 | 93.9 | 56.1 | 53.6 | 152.9 | |
| Gunma | 23.5 | 4.8 | 69.0 | 53.8 | 55.6 | 161.2 | |
| Saitama | 20.9 | 7.4 | 32.9 | 39.9 | 26.6 | 81.7 | |
| Chiba | 21.7 | 6.5 | 43.9 | 46.7 | 32.8 | 68.8 | |
| Tokyo | 19.4 | 4.3 | 47.7 | 56.8 | 68.6 | 85.0 | |
| Kanagawa | 19.0 | 4.8 | 33.1 | 39.0 | 37.2 | 54.4 | |
| Niigata | 25.4 | 5.8 | 84.6 | 32.9 | 42.3 | 59.0 | |
| Toyama | 22.5 | 5.4 | 71.5 | 49.8 | 48.4 | 106.4 | |
| Ishikawa | 21.9 | 5.6 | 68.4 | 54.7 | 56.2 | 123.7 | |
| Fukui | 17.9 | 6.6 | 55.1 | 74.7 | 53.6 | 165.8 | |
| Yamanashi | 23.0 | 6.8 | 66.3 | 56.4 | 52.1 | 141.7 | |
| Nagano | 22.2 | 5.9 | 71.4 | 37.4 | 71.2 | 188.4 | |
| Gifu | 19.8 | 7.8 | 45.3 | 41.5 | 38.2 | 75.9 | |
| Shizuoka | 23.3 | 5.6 | 52.1 | 38.8 | 47.6 | 93.3 | |
| Aichi | 20.7 | 5.4 | 39.6 | 41.1 | 43.2 | 80.7 | |
| Mie | 23.1 | 7.6 | 34.3 | 41.1 | 41.3 | 73.4 | |
| Shiga | 17.1 | 8.1 | 33.1 | 36.7 | 26.7 | 50.3 | |
| Kyoto | 18.8 | 4.9 | 37.0 | 44.5 | 50.8 | 87.0 | |
| Osaka | 16.6 | 5.0 | 27.3 | 42.3 | 38.8 | 67.3 | |
| Hyogo | 19.1 | 7.6 | 26.0 | 37.7 | 37.4 | 60.5 | |
| Nara | 17.8 | 4.1 | 24.5 | 33.6 | 31.1 | 45.6 | |
| Wakayama | 19.5 | 7.9 | 34.7 | 33.7 | 32.3 | 50.1 | |
| Tottori | 22.9 | 10.7 | 55.4 | 35.2 | 31.9 | 56.4 | |
| Shimane | 21.3 | 4.3 | 58.4 | 31.0 | 26.5 | 93.6 | |
| Okayama | 21.0 | 9.6 | 48.0 | 35.4 | 29.7 | 82.6 | |
| Hiroshima | 19.0 | 6.9 | 58.7 | 33.0 | 38.5 | 100.4 | |
| Yamaguchi | 21.2 | 4.9 | 43.6 | 35.0 | 24.5 | 70.7 | |
| Tokushima | 20.1 | 4.9 | 65.6 | 29.3 | 26.5 | 107.4 | |
| Kagawa | 18.1 | 6.0 | 37.5 | 30.3 | 31.1 | 200.8 | |
| Ehime | 20.0 | 4.4 | 45.4 | 43.4 | 34.3 | 77.9 | |
| Kochi | 20.2 | 9.8 | 44.8 | 22.0 | 29.3 | 61.5 | |
| Fukuoka | 17.7 | 4.6 | 60.6 | 38.2 | 41.5 | 80.7 | |
| Saga | 20.7 | 3.5 | 81.7 | 48.3 | 34.8 | 100.2 | |
| Nagasaki | 19.3 | 8.1 | 53.2 | 36.3 | 31.3 | 77.0 | |
| Kumamoto | 19.2 | 3.5 | 69.1 | 36.1 | 33.6 | 65.8 | |
| Oita | 18.8 | 7.5 | 53.5 | 44.8 | 36.0 | 91.8 | |
| Miyazaki | 26.3 | 6.2 | 69.0 | 38.7 | 37.2 | 98.8 | |
| Kagoshima | 27.5 | 9.2 | 72.7 | 36.9 | 36.9 | 80.2 | |
| Okinawa | 17.5 | 5.7 | 75.4 | 54.6 | 38.7 | 65.6 | |

AMI: acute myocardial infarction
